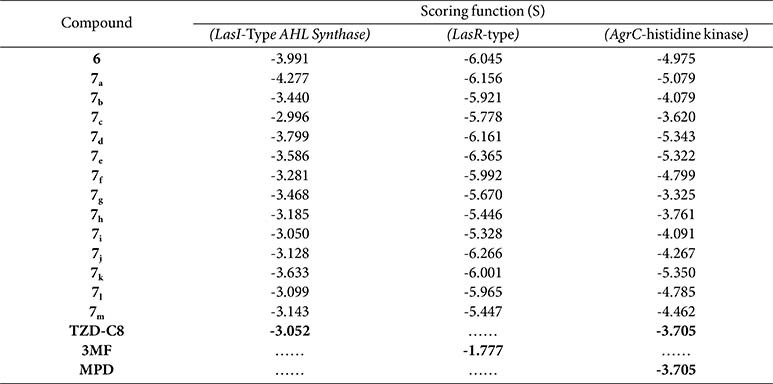# Disclosing New Insights on Pyrazolo[3,4-*d*] Pyrimidine Tethered Diverse Amino Acid Candidates as Potential DHFR Inhibitors and Anti-Virulence Agents

**DOI:** 10.4014/jmb.2508.08055

**Published:** 2025-12-11

**Authors:** Tarek S. Ibrahim, Ibrahim M. Salem, Nabil A. Alhakamy, Amany M. M. Al-Mahmoudy, Wael A. H. Hegazy

**Affiliations:** 1 Department of Pharmaceutical Chemistry, Faculty of Pharmacy, King Abdulaziz University, Jeddah 21589, Saudi Arabia; 2 Pharmaceutical Chemistry Department, College of Pharmacy, Al-Farahidi University, Baghdad 10070, Iraq; 3 Department of Pharmaceutics, Faculty of Pharmacy, King Abdulaziz University, Jeddah 21589, Saudi Arabia; 4 Center of Excellence for Drug Research and Pharmaceutical Industries, King Abdulaziz University, Jeddah 21589, Saudi Arabia; 5 Mohamed Saeed Tamer Chair for Pharmaceutical Industries, King Abdulaziz University, Jeddah 21589, Saudi Arabia; 6 Department of Pharmaceutical Organic Chemistry, Faculty of Pharmacy, Zagazig University, Zagazig 44519, Egypt; 7 Department of Microbiology and Immunology, Faculty of Pharmacy, Zagazig University, Zagazig 44519, Egypt; 8 Pharmacy Program, Department of Pharmaceutical Sciences, Oman College of Health Sciences, Muscat 113, Oman

**Keywords:** DHFR inhibitors, bacterial resistance, quorum sensing, anti-virulence, *Pseudomonas aeruginosa*, *Staphylococcus aureus*, healthcare

## Abstract

The rise of bacterial resistance presents a pressing public health challenge, necessitating innovative antimicrobial solutions. Based on our prior work demonstrating the activity of pyrazolo[3,4-*d*] pyrimidine-based analogues against human dihydrofolate reductase (DHFR), we hypothesized that novel derivatives could function as dual-action antibacterial agents. In this study, we therefore evaluated the capacity of these compounds to both inhibit bacterial DHFR and disrupt quorum sensing (QS)-mediated virulence. The compounds were screened for antibacterial activity against several bacterial strains and the anti-DHF activity was assayed. Anti-virulence potential was assessed *in vitro* and *in vivo*, while interactions with the QS targets were investigated using in silico study. *Staphylococcus aureus* and *Pseudomonas aeruginosa* were chosen as representative clinically important gram-negative and gram-positive pathogens, respectively, to evaluate the antibacterial and anti-virulence activities. The *in vitro* and in silico DHFR inhibition assays revealed potent antibacterial activity for the synthesized compounds against various bacteria. Among the most promising candidates, compound 7_e_ exhibited potent antibacterial activity at low minimum inhibitory concentrations (MICs) and demonstrated synergy with conventional antibiotics. The *in vitro* and *in vivo* evaluations showed promising anti-virulence activities of the synthesized compounds, particularly 7_a_ and 7_e_, against *P. aeruginosa* and *S. aureus*, respectively. Dynamics simulations showed the strong binding affinity of 7_a_ and 7_e_ to LasI/R and AgrC QS targets in *P. aeruginosa* and *S. aureus*, respectively. In conclusion, our findings demonstrate that pyrazolo[3,4-d]pyrimidine analogues, especially 7_a_ and 7_e_, can function as effective dual-action agents by inhibiting DHFR and suppressing QS-mediated virulence, thus representing a promising new class of anti-virulence therapeutics against priority bacterial pathogens.

## Introduction

Dihydrofolate reductase (DHFR) is the enzyme that catalyzes the terminal step of tetrahydrofolate (THF) synthesis through the reduction of the dihydrofolate (DHF) precursor. DHFR enzyme inhibition ensures great antibacterial activity as it results in significant repression of the de novo biosynthesis of nucleotides, thereby diminishing the proper DNA replication capability of bacterial cells as a consequence [1]. The inhibition of DHFR holds significant value in treating bacterial and protozoal infections [2, 3]. Trimethoprim (TMP), a well-known antibiotic and potent inhibitor of bacterial DHFR, is widely used to treat respiratory tract infections, urinary tract infections, and other bacterial diseases [4]. By selectively targeting bacterial DHFR while sparing human DHFR, **TMP** exhibits high efficacy with relatively low toxicity to the host [5, 6]. In addition to DHFR in microbial pathogens, the enzyme is also targeted in cancer therapy, and methotrexate (MTX), a DHFR inhibitor, is commonly used to inhibit the growth of rapidly dividing cancer cells. By disrupting folate metabolism in cancer cells, MTX effectively halts cell proliferation and induces apoptosis [7]. However, despite the success of DHFR-targeting drugs, the emergence of resistance poses a significant challenge. Mutations in the DHFR-encoding genes, namely the *fol* genes, can lead to reduced drug binding affinity, rendering DHFR inhibitors less effective [8]. Therefore, ongoing research aims to develop new DHFR inhibitors with improved efficacy and reduced susceptibility to resistance mechanisms.

Quorum sensing (QS) is a sophisticated communication system used by both gram-positive and gram-negative bacteria to control virulence gene expression in response to variations in cell population density [9]. Through QS, bacteria can coordinate collective behaviors, such as virulence factor production, biofilm formation, and the expression of antibiotic resistance mechanisms [10, 11]. The QS process typically involves the production and secretion of signaling molecules called autoinducers [12, 13]. As the bacterial population density increases, the concentration of these autoinducers rises. Once a threshold concentration is reached, the autoinducers bind to specific receptors, initiating a cascade of signaling events that ultimately regulate gene expression [9, 14]. Targeting QS has arisen as an interesting approach for the development of novel antimicrobial therapies [15-17]. By disrupting QS, it is possible to interfere with bacterial communication and inhibit the expression of virulence factors essential for pathogenesis [15, 18, 19]. This approach offers several potential advantages over traditional antibiotics, including reduced selective pressure for resistance development and the ability to attenuate bacterial pathogenicity without directly killing the bacteria [20, 21]. Natural compounds derived from plants, animals, and microorganisms have shown promise as QS inhibitors [22-24]. Additionally, synthetic compounds designed to specifically target QS components have been developed and tested for their antimicrobial activity [25-28].

Sulfonamides competitively inhibit dihydropteroate synthase (DHPS), encoded by the *folP* gene, while the *folA* gene encodes DHFR, which could be targeted by **TMP** [1]. Interestingly, QS has been proposed to govern the expression of *fol* genes within bacterial cells. Conversely, the suppression of QS may potentially trigger an upsurge in the metabolic requirement for folate [29], which suggests the potential impact of antifolate drugs with anti-QS activity. Therefore, we sought to assess the antibacterial and anti-virulence activities of our synthesized DHFR-inhibiting compounds.

**Fig. 1:**
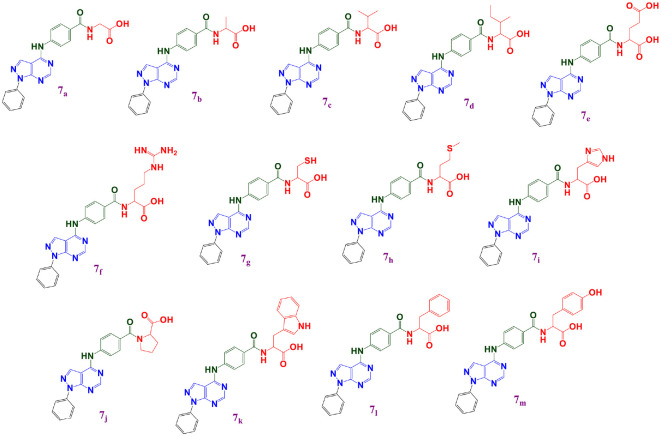
The chemical structures of the proposed pyrazolo[3,4-*d*]pyrimidines (7_a-m_) incorporating different amino acid conjugates, as DHFR inhibitors and promising antivirulence agents.

Recently, we reported novel, DHFR-inhibiting pyrazolo[3,4-*d*] pyrimidine scaffolds **7_a-m_** bearing a 4-aminobenzyl group and conjugated with a series of different amino acids. The investigated compounds **7_a-m_** were prepared according to reported procedures [30]. Coincidentally, the compounds revealed promising antitumor behavior superior to the antineoplastic drug MTX (Fig. 1) [30]. The antimicrobial activity of pyrazolo[3,4-*d*]pyrimidine-based candidates was frequently reported, with distinctive MIC and IC_50_ values against various bacterial strains [31-33]. Whereas the pyrazolo[3,4-*d*]pyrimidine bearing thienyl hydrazone compound **I** revealed noteworthy IC_50_ results against *S. aureus*, *P. aeruginosa*, and *E. coli* [31], the pyrazolo[3,4-*d*]pyrimidine compound **II** also exerted prominent effects, especially against *E. coli*, achieving an antibacterial concentration of 50 μg/ml [33]. Furthermore, the reported pyrazolo[3,4-*d*]pyrimidine incorporating pyrazole compound **III** showed substantial MIC values of 0.24 and 0.06 μg/ml against *P. aeruginosa* and *E. coli* respectively [32].

**Fig. 2:**
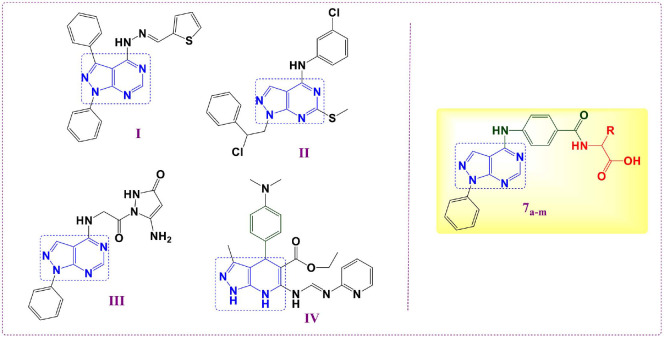
Chemical architecture represents the structure similarity of the reported antivirulence pyrazolo[3,4-*b*]pyridines I and II besides the inspected pyrazolo[3,4-*d*]pyrimidines (7_a-m_).

The anti-virulence activity of the evaluated pyrazolo[3,4-*d*]pyrimidine derivatives was expected since they are considered the classical ring equivalent bioisosteres of the recently reported QS inhibitor pyrazolo[3,4-*b*]pyridine compound **IV** (Fig. 2) [34]. Due to prior outcomes, we estimated the DHFR inhibition at the MICs of the inspected pyrazolo[3,4-*d*]pyrimidines **7_a-m_**, measured against various gram-negative and gram-positive strains, as an indication of their antibacterial potential. Furthermore, we assessed the *in vivo* and *in vitro* anti-virulence activities of the candidate compounds to confirm their inhibitory activity against the resistant bacterial strains, and their worthiness for further investigation.

## Materials and Methods

### Detection of Minimum Inhibitory Concentrations (MICs)

Cultures of *S. aureus* (ATCC 6538), *P. aeruginosa* (ATCC 47085), *Staphylococcus epidermidis* (ATCC 12228), *Micrococcus luteus* (ATCC 10240), *Klebsiella pneumoniae* (ATCC 27736), *Salmonella* Typhimurium (ATCC 14028) and *E. coli* (ATCC 10536) were freshly prepared by growing them in Mueller-Hinton broth to an approximate density of 1 × 10^6^ CFU/ml. The MICs of the synthesized compounds or tested antibiotics (amoxycillin, ciprofloxacin and sulfamethoxazole/trimethoprim) against the bacterial strains were evaluated using the broth microdilution technique in accordance with the Clinical Laboratory and Standards Institute (CLSI, 2020) [35, 36](detailed description provided in Supplementary Materials).

### DHFR Inhibitor Assay

A Dihydrofolate Reductase Inhibitor Screening Kit (Cat. No. ab283374, Abcam, USA) was used to screen the DHFR inhibitors. Positive and negative controls and compound samples (at MIC concentrations) were prepared according to the protocol provided by the manufacturer. The activity of DHFR was observed by the decrease in absorbance at OD 340 nm, while potential inhibitors could arrest this decrease (detailed description provided in Supplementary Materials).

### Checkerboard Method to Determine the Potency of Combination

The combination of synthesized compounds or tested antibiotics (amoxycillin, ciprofloxacin and sulfamethoxazole/trimethoprim) was assessed using the checkerboard method as previously detailed [10, 37]. Briefly, twofold serial dilutions of synthesized compounds and antibiotics were executed in a 96-well plate. Subsequently, 100 μl of *S. aureus* and *P. aeruginosa* overnight cultures (OD_600_ 0.4) were added to each well. The absence of visible turbidity at the lowest concentrations was deemed the MICs of the combination. The Fractional Inhibitory Concentration (FIC) was computed using the formula FIC = combined MIC of antibiotic/MIC of antibiotic + combined MIC of compound/MIC of compound. Synergism was considered when FIC ≤ 0.5, an additive effect for 0.5 < FIC ≤ 1, no effect for 1 < FIC ≤ 2, and an antagonistic effect for FIC > 2 [37].

### Bacterial Viable Counts

Loopfuls of fresh *S. aureus* and *P. aeruginosa* cultures were suspended in normal physiological saline (0.9%sodium chloride solution), and their turbidities were adjusted to an OD_600_ of 0.4. The viable bacterial cells were counted in the presence and absence of the tested compounds at sub-MIC (1/2 MIC) to assess their impact on bacterial growth as previously shown [38, 39] (detailed description provided in Supplementary Materials).

### Biofilm Formation Evaluation

The biofilm formation assay was conducted by employing the crystal violet (CV) method as described previously [40-42]. The OD of overnight *S. aureus* and *P. aeruginosa* cultures, supplemented with or without the tested compounds at 1/2 MIC, was adjusted to an OD_600_ of 0.4. Aliquots (200 μl) of bacterial suspensions were added into sterile, 96-well microtiter plates and incubated at 37°C for 24 h. The planktonic bacteria were removed, and each underwent two rinses with phosphate-buffered saline (PBS). The plates were allowed to air-dry before being stained by adding 1% CV solution prepared in 100% ethanol, and the plates were then incubated for 20 min. The excess CV was removed, and the plates were rinsed with sterile PBS. After air drying, CV was solubilized by 30%glacial acetic acid to measure the optical densities at OD_590_. Positive and negative controls, represented by sub-MICs of ciprofloxacin and 1% v/v dimethyl sulfoxide (DMSO), respectively, were employed in the experiment.

### Proteases Assay

The protease production was assayed in the presence or absence of the selected compounds at 1/2 MIC [22, 43]. Overnight *S. aureus* and *P. aeruginosa* cultures grown with or without the tested compounds at 1/2 MIC were normalized to an OD_600_ of 0.4. The supernatants were then collected, mixed in a 1:1 ratio with 2% casein (pH 7.0), and incubated at 37°C for 25 min. The reaction was then stopped by adding 2 ml of trichloroacetic acid (0.4 M) for 25 min, and the OD of the supernatants was assessed at 660 nm.

### Hemolysins Assay

The hemolysin production was tested in the presence or absence of the selected compounds at 1/2 MIC as described [44, 45]. The supernatants were collected as described above, mixed with 2% erythrocyte suspension (1:1 ratio), and kept for 2 h at 37°C. The OD was measured at 540 nm. Negative and positive controls were carried out using un-hemolyzed erythrocytes and fully hemolyzed erythrocytes with 0.1% sodium dodecyl sulfate.

### Mice Survival Assay

The *in vivo* protection assay was performed to assess the capacity of tested compounds at 1/2 MIC to lessen the *P. aeruginosa* and *S. aureus* pathogenesis [41, 43, 46, 47]. The fresh bacterial cultures were prepared and grown in the presence or absence of the selected compounds at 1/2 MIC (OD_600_ 0.4). A total of six groups, each consisting of 10 three-week-old mice (*Mus musculus*), were established for the experiment. The first and second groups functioned as negative controls and did not undergo either inoculation or intraperitoneal (IP) injections; instead, they received sterile PBS injections. The third and fourth groups were IP injected with DMSO-treated *P. aeruginosa* or *S. aureus*, serving as positive controls. The fifth group was IP injected with *P. aeruginosa* treated with compound **7_a_** at 1/2 MIC. The sixth group was injected with *S. aureus* treated with compound **7_e_** at 1/2 MIC. The survival of the mice was observed over six consecutive days, and death was recorded using The Kaplan–Meier method. A log-rank test for trend was employed to determine the statistical significance of a tested compound's ability to reduce bacterial virulence.

### Ligand Construction, Target Preparation, Receptor Grid Generation, and Docking Protocol

Molecular docking deliberations of the most stable pose of the most active antibacterial pyrazolo[3,4-*d*]pyrimidine compound, **7_e_**, were executed against the *E. coli* DHFR biotarget (PDB ID: 4RGC) [48] in relation to the reference drug trimethoprim (TMP), using Schrodinger’s Maestro (Version 2024-1). Meanwhile, all of the inspected pyrazolo[3,4-*d*]pyrimidine candidates (**6** and **7_a-m_**) were docked against different biotargets using the same software, comparable with the certified native ligands, to ensure their discriminatory binding affinity and to suppose their anti-virulence effect. The selected biotargets were: *P. aeruginosa* virulence targets LasI-synthetase (PDB ID: 1RO5) [49] and LasR-type (PDB ID: 6MVN) [50, 51], and *S. aureus* virulence regulator ArgC histidine kinase ATP-binding domain (PDB ID: 4BXI) [52]. The most stable poses of the designated compounds and the authenticated reference ligands were constructed using Chemdraw 18.0 PerkinElmer software and cleaned up for bond alignment before being imported to the Schrodinger software in a 3D representation fashion. The energy minimization was carried out using the OPLS3e (Optimized Potentials for Liquid Simulations) [53] force field in LigPrep (Version 2024-1, Schrodinger) [54]. This minimization facilitates appointing the bond orders and the addition of hydrogen atoms to the explored hits. The established output files that denote the favorable conformers of the ligands were further used for docking consideration. Also, the chosen targets were exposed to certain preparations using the Protein Prep Wizard (Version 2024-1, Schrodinger) [55] as a main implement for protein preparation. Protonation was carried out on the proteins, and the charges were authorized using Epik at pH 7.0 ± 2.0. The target proteins were exposed to pre-processing, refinement, and protein modification by the relevant chain selection and water molecule removal, and to finish, the protein was minimized using the OPLS3 force field. Otherwise, the receptor grids were created by considering the co-crystal ligands (X-ray pose of the ligand in the protein) for all biotargets, except for *P. aeruginosa* LasI-synthetase (PDB ID: 1RO5), due to the absence of the native ligand, where a grid box was created via residue selection of the largest binding pocket residues. For the remainder of the scrutinized biotargets, the centroid of the ligands was selected to create a grid box around it, and the Van der Waals radius of the receptor atoms was scaled to 10 Å with a partial atomic charge of 0.25. The molecular docking was accomplished with a flexible docking protocol [56] by using Schrodinger’s Maestro software. All the docking computations were run using Extra Precision (XP) mode, in which a scaling factor of 0.8 and a partial atomic charge < 0.15 were involved with the protein atoms. Glide docking scores were preserved to verify the best-docked poses from the output, and the interactions of these docked poses were further examined using XP visualizer. Following the last step of docking with the co-crystal ligand in XP mode, the root mean square deviation (RMSD) values that lay within 0.46 Å were certified to validate the protein.

Lastly, Lipinski’s rules for all of the investigated pyrazolo[3,4-*d*]pyrimidines (**6** and **7_a-m_**), including molecular weights (≤ 500), LogP (≤ 5), HBD (≤ 5), and HBA (≤ 10), were calculated using Molecular Operating Environment (MOE) Suite V.2014.09 to confirm the oral aptitude and capability of each compound as a drug worthy of further investigation.

### Molecular Dynamic Simulation

We appraised the stability of our promising compounds using molecular dynamic (MD) simulations on the OPLS3e force field with the Desmond software (Desmond 2024–1, Schrodinger). The evaluated compounds were pyrazolo[3,4-*d*]pyrimidine compound **7_e_**, along with the reference drug **TMP** in complex with the *E. coli* DHFR active site, in addition to the most active anti-virulence compounds, namely **7_a_** against *P. aeruginosa* in complex with the active sites of the LasI-synthetase and LasR-type related to the native ligands **TZD-C8** and **3MF**, and compound **7_e_** against *S. aureus* in complex with the AgrC histidine kinase active site comparable with the **MDP** native ligand [57]. The ligand–protein complex was fixed in a solvent-soaked orthorhombic periodic box with a minimum distance of 10 Å between protein atoms and the box edges. The solvent was fulfilled via a single point charge (OPLS3) water model. The system charge was neutralized by integrating Na^+^ and Cl^-^ counter ions and 0.15 M NaCl salt concentration was set consistent with the physiological system using the Desmond *System Builder* panel [58]. Afterward, the assembled solvated system was minimized and relaxed using OPLS4e force field constraints as the default protocol associated with Desmond [53]. The isothermal isobaric collaborative (normal pressure and temperature/NPT) was established during the simulation, with a temperature of 300k and an atmospheric pressure of 1.0315 bar using the Nose-Hoover chain thermostat method and the Martyna-Tobias-Klein barostat method, respectively [59, 60]. The simulation was run for a period of 100 ns and the MD trajectories were scrutinized using Desmond’s Simulation Interaction Diagram (SID) to forecast the binding orientation of the ligand. The generated MD simulation trajectories were used to compute several considerations, including the protein Cα atoms’ root mean square deviation (RMSD), the RMSD of ligand atoms, protein root mean square fluctuation (RMSF), and protein ligand contact histogram mapping. Speculation of the RMSD in the MD simulations gives an estimate of the stability of the protein–ligand complexes and their dynamic behavior. The deviation in the structure of a protein or protein–ligand complex from their initial poses is considered to be an RMSD, and accounts for the stability of the protein–ligand complex throughout the duration of the simulation [61].

## Results and Discussion

### Antibacterial Activity

**MICs of the synthesized compounds against various gram-negative and gram-positive bacteria.** The antibacterial activity of the tested compounds was assessed by determining their MICs against representative gram-negative (*P. aeruginosa*, *K. pneumoniae*, *Salmonella* Typhimurium, and *E. coli*) and gram-positive (*S. aureus*, *S. epidermidis*, *M. luteus*) strains. Several compounds exhibited lower MIC values in comparison to the used reference drug trimethoprim-sulfamethoxazole (SXT) as shown in Table 1. In compliance with the docking scores of the tested compounds into DHFR, compounds **6** and **7_f_** showed the highest MIC values, indicating low antibacterial activity. On the other hand, compounds **7_c-e_**, **7_g_**, **7_h_**, and **7_k-m_** showed considerable antibacterial activity against the tested strains.

### Screening of the DHFR Inhibition

**Fig. 3:**
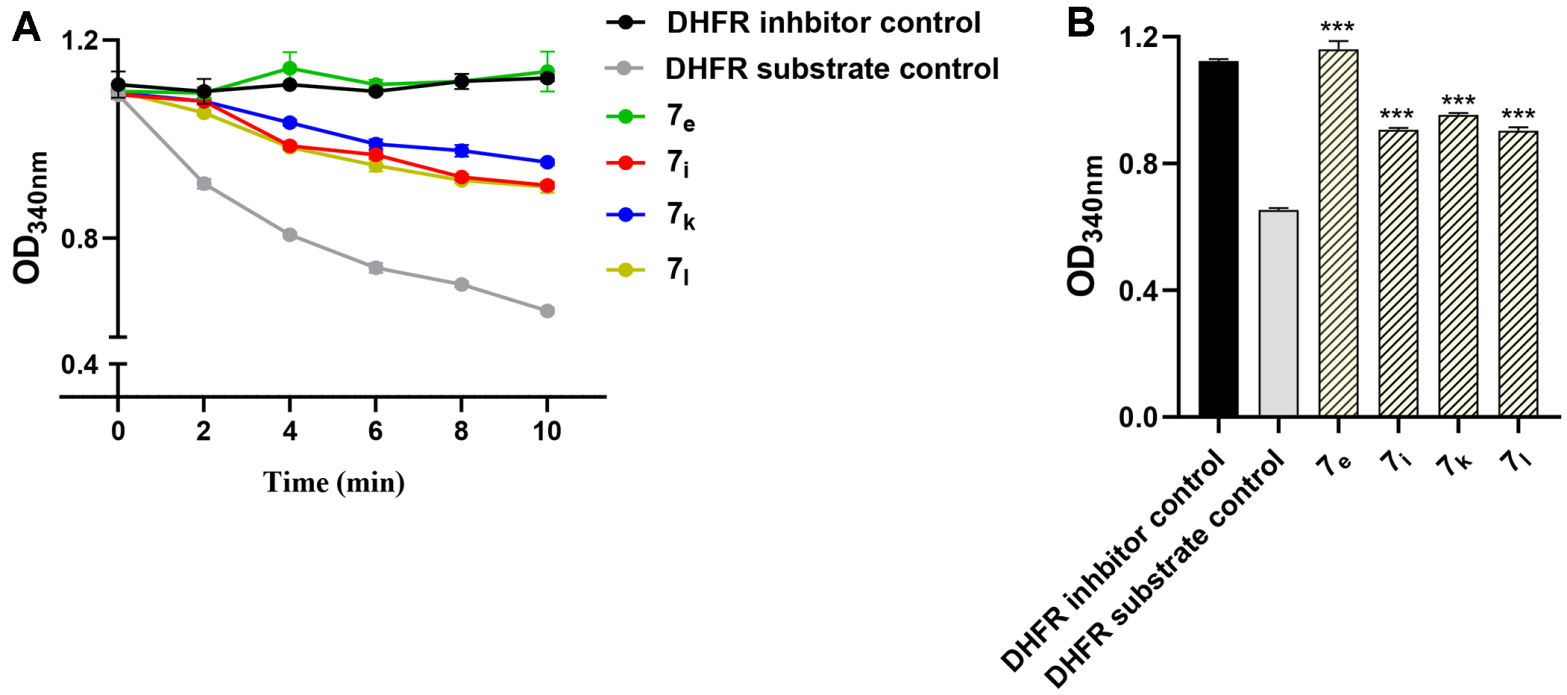
DHFR Inhibitor Screening. (**A**) DHFR activity is observed through the reduction in absorbance and potential inhibitors prevent this decrease through different time points. (**B**) The activity of DHFR at the end of the experiment (after 10 min). The current results showed significant inhibition of tested compounds to the DHFR enzyme, especially **7_e_**. ***: *p* < 0.001.

To evaluate the DHFR inhibition of the tested compounds, a colorimetric assay was conducted. The selection of compounds **7_e_**, **7_i_**, **7_k_**, and **7_l_** for DHFR activity assessment was based on their potent antibacterial activity, as evidenced by their low MIC values against the tested gram-positive and gram-negative pathogens (Fig. 3). DHFR activity is assessed by observing the decrease in absorbance at OD 340 nm, with potential inhibitors halting this reduction. While the absorbance significantly decreased when adding the DHFR substrate, the absorbance was not decreased by applying the DHFR inhibitor control. Interestingly, the tested compounds significantly increased the absorbance in comparison to the control, particularly **7_e_**, indicating their inhibition of DHFR. The test was replicated in triplicates, and statistical significance was evaluated employing a two-way repeated measures ANOVA.

### Synergistic Outcome of 7_e_ Combination with Antibacterials

**Fig. 4:**
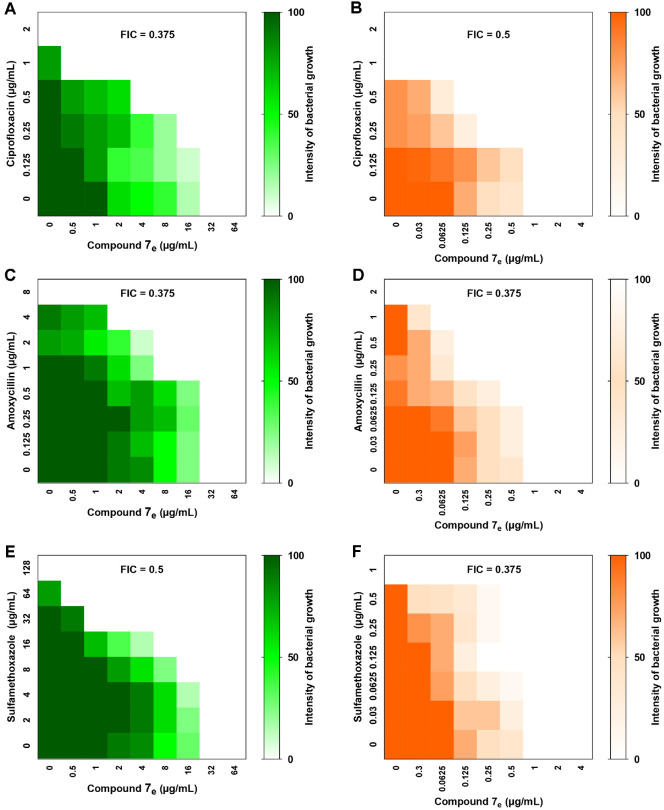
Compound 7_e_ showed synergistic outcomes when combined with (A, B) ciprofloxacin, (C, D) amoxicillin, and (E, F) sulfamethoxazole against *P. aeruginosa* and *S. aureus*, respectively. The FICs values were ≤ 0.5 which indicates a synergistic outcome.

To attest to the antimicrobial activity of compound **7_e_**, the checkerboard method was used to estimate the outcome of a combination with amoxicillin, ciprofloxacin, and sulfamethoxazole. Initially, the MIC values of the tested antibacterial agents were assessed against *S. aureus* and *P. aeruginosa*. The MICs of amoxicillin, ciprofloxacin, and sulfamethoxazole/trimethoprim were 8 μg/ml, 2 μg/ml, and 64 μg/ml against *P. aeruginosa*, respectively. The MICs against *S. aureus* were 2 μg/ml, 1 μg/ml, and 1 μg/ml for amoxicillin, ciprofloxacin, and sulfamethoxazole, respectively. By combining the tested antibacterial agents with compound **7_e_**, a considered decrease in MIC values was observed, and the FIC values were always less than 0.5, indicating a synergistic outcome (Fig. 4).

### Docking Analysis of the Synthesized Compounds into the DHFR Binding Site

**Fig. 5:**
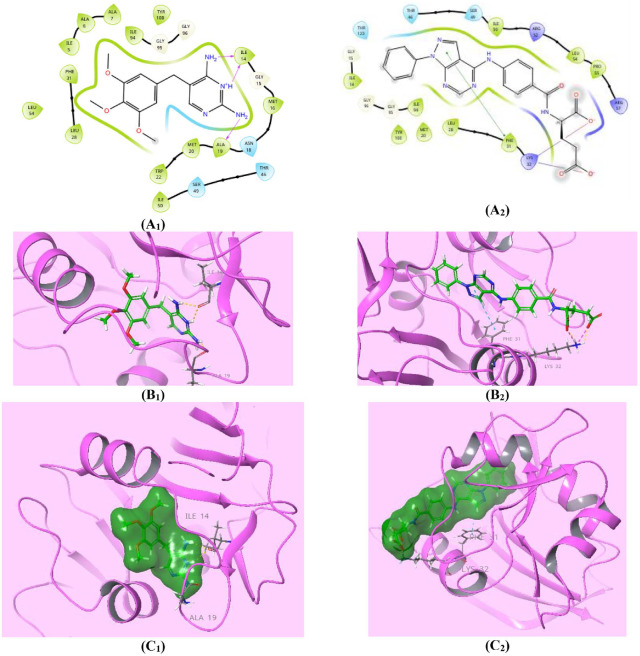
Compound 7_e_ showed competitive affinity to DHFR. (**A_1,2_**) Two-dimensional structural representation of the reference ligand trimethoprim (TMP) and the investigated pyrazolo[3,4-*d*]pyrimidine derivative **7_e_** in the active site of *E. coli* DHFR enzyme (pdb ID: 4RGC). (**B_1,2_**) Three-dimensional representation of the *E. coli* DHFR binding site with an overlay of the reference ligand trimethoprim (TMP) and the pyrazolo[3,4-*d*]pyrimidine derivative **7_e_** (green sticks). (**C_1,2_**) Threedimensional cartoon representations of the *E. coli* DHFR binding domain co-crystallized with the reference ligand trimethoprim (TMP) and the pyrazolo[3,4-*d*]pyrimidine derivative **7_e_** (green space-filling).

DHFR is an essential enzyme responsible for catalyzing the NADPH-dependent reduction of 7,8-dihydrofolate (DHF) to 5,6,7,8-tetrahydrofolate (THF) [62]. This enzymatic reaction is crucial for various cellular processes, including nucleotide synthesis, amino acid metabolism, and several mainly one-carbon transfer reactions to generate methionine, thymidine, serine, glycine, and other biological molecules [63]. Therefore, depressing DHFR is an expedient target in the development of prominent antimicrobial drugs, such as **TMP**, cycloguanil, and pyrimethamine (PYR) [64]. The requirements for the reduction catalysis mechanism of DHFR through the direct protonation of the DHF substrate by the solvent were: conformational disorder of the Met20 residue side chain and high electron density at the N5 atom of the folate pteridine ring beside a partially occupied water molecule within hydrogen-bonding distance of the N5 atom of the folate substrate, clarified using the 1.05-Å resolution X-ray structure of the ternary complex of *E. coli* DHFR with folate and NADP^+^ [48, 65]. Accordingly, restraining any of these requirements produces perspicuous retardation of the reduction catalysis mechanism of the DHFR enzyme [48]. In this study, since pyrazolo[3,4-*d*]pyrimidine derivatives **6** and **7_a-m_** have pellucid inhibition activity against DHFR enzyme [30], molecular docking investigations were performed for the most active pyrazolo[3,4-*d*]pyrimidine bearing glutamic acid compound **7_e_**, as well as for the **TMP** reference drug, within the *E. coli* DHFR active site for enriched insights about the differential DHFR inhibition activities. Auspiciously, compound **7_e_** (*S* = -5.898 Kcal/mol) disclosed superior affinity over **TMP** (*S* = -5.409 Kcal/mol) during virtual docking simulation. Both compounds, **7_e_** and **TMP**, fit the active site of the DHFR in a comparable pose through high exposure to both conformations of the crucial Met20 residue, which explains how these compounds mediate the favorable inhibition activity of the catalysis function of the DHFR enzyme (Fig. 5). Furthermore, the higher binding energy assigned for compound **7_e_** may be due to the lucid ionic bonding of both α- and γ-carboxylate moieties of glutamic acid with Lys32 residue, with bond lengths of (2.63A^o^) and (2.90A^o^) respectively, as well as the worthy π-π* stacking between the pyrazole core and the crucial Phe31 residue (5.37A^o^) (Fig. 5A_2_, B_2_, C_2_). Whereas, the binding affinity of **TMP** could be elucidated by two prominent H-bond donor effects of two amino groups of the pyrimidine core with Ile14 (2.07A^o^) and Ala19 (2.19A^o^) residues, along with another characteristic H-bond donor effect of a protonated N_3_ of the pyrimidine ring with the Ala19 amino acid (2.38A^o^) (Fig. 5A_1_, B_1_, C_1_). Lastly, compound **7_e_** is forecasted to exert a marked antibacterial effect against *E. coli* through its potential inhibition of the DHFR enzyme, although further study is needed for confirmation.

In terms of profitability, all synthesized compounds adhere to Lipinski’s rule, except for compound **7_f_**. This deviation is attributed to the presence of the guanidine moiety derived from the arginine amino acid, which results in an increased number of hydrogen bond donor (HBD) groups to **7**, and the number of hydrogen bond acceptor (HBA) groups to 12, within the compound, thereby explaining the drug-like capabilities of compound **7_e_** (Table 2).

### Molecular Dynamic Simulation of Compound 7_e_ in Complex with the DHFR Biotarget Related to Trimethoprim (TMP)

**Fig. 6:**
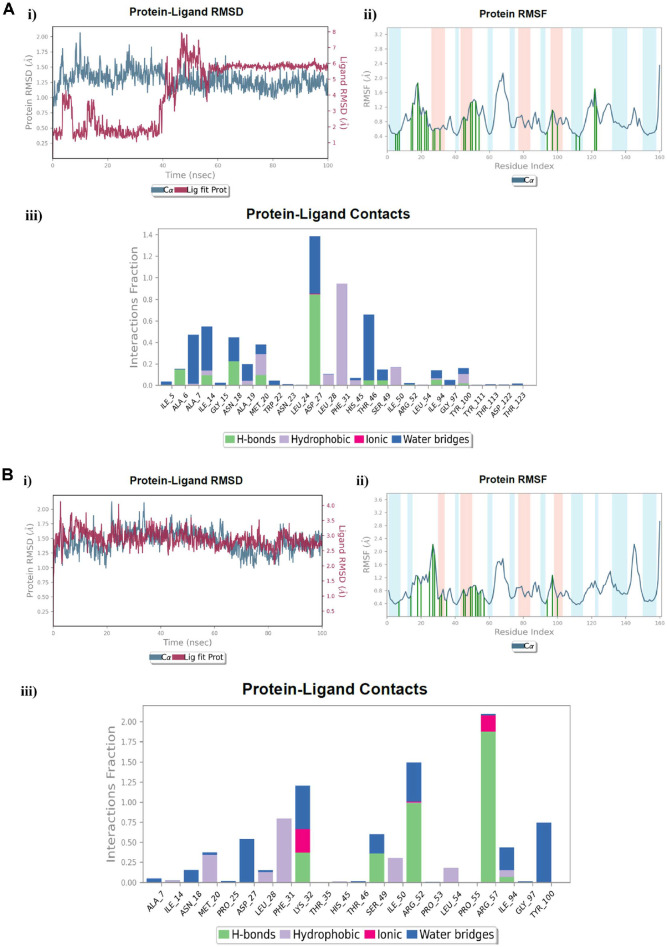
MD simulation analysis of; (**A**) The reference drug **TMP** in complex with the DHFR active site, (**B**) The investigated compound **7_e_** in complex with the DHFR active site, **i**) RMSD (Protein RMSD is shown in grey while the RMSD of the **TMP** and **7_e_** ligands is shown in red), **ii**) Protein RMSF, **iii**) Protein–ligand contact histogram analysis of the MD trajectory.

We utilized the MD simulation technique in a certain hydration environment for 100 ns to consider the dynamic performance of the DHFR active site in complex with the **TMP** reference drug, and the investigated pyrazolo[3,4-*d*]pyrimidine incorporating glutamic acid compound **7_e_**, as well as to authorize the stability of the docking complex between them and the DHFR protein (PDB ID: 4RGC). Fortunately, converging the protein RMSD analysis of the DHFR complex with **TMP** and compound **7_e_**, the Cα atom variations were steadily within 2.1 Å throughout the simulation, with regular fluctuations starting from approximately 0.8 Å (Fig. 6A, 6B: i). We providentially observed that none of the RMSD fluctuations were outside the acceptable limit of 3 Å. Nevertheless, during simulation of the DHFR-**7_e_** complex, modest fluctuations were observable during the simulation, but these stabilized within the simulation period (Fig. 6B: i). After the preliminary fluctuation outstanding to equilibration, the RMSD of the **TMP** and **7_e_** ligands during complexation with the protein system were firmly between 3.0 Å and 8.0 Å, and from 2.0 Å to 4.2 Å, respectively, throughout the simulation, thereby reflecting the favorable binding of compound **7_e_** compared to the **TMP** reference drug with fewer fluctuations. Furthermore, constraint of the RMSF was accomplished to explore each residue fluctuation and the conformational changes within the protein chain during the simulation. If the main active site residues fluctuated very slightly, then there were minor conformational alterations, denoting the firm binding of the investigated compound inside the active site [66]. In the protein RMSF design, the α-helices and β-strand residues were represented on red and blue backgrounds, respectively, although the loop region was depicted on a white one. Commonly, the residues of the α-helices and β-strands fluctuated less than the loop regions, whereas the major fluctuations were kept within the loop region [67]. Moreover, the involvement of the combined residues of the protein chain and each ligand was depicted by vertical green lines on the plot’s *x*-axis, where the reference drug **TMP** contacted 27 residues of the DHFR protein, with most of them being in the highly fluctuated loop region. These residues were namely Ile5, Ala6, Ala7, Ile14, Gly15, Asn18, Ala19, Met20, Trp22, Asn23, Leu24, Asp27, Leu28, Phe31, His45, Thr46, Ser49, Ile50, Arg52, Leu54, Ile94, Gly97, Tyr100, Tyr111, Thr113, Asp122, and Thr123 (Fig. 6A: ii). Perceptibly, in the DHFR-**7_e_** complex, we noticed that it could bind to most of the combined residues with the **TMP** compound, with the exception of 11 residues, namely Ile5, Ala6, Gly15, Ala19, Trp22, Asn23, Leu24, Tyr111, Thr113, Asp122, and Thr123. The complex exerted additional binding to the 6 residues Pro25, Lys32, Thr35, Pro53, Pro55, and Arg57 (Fig. 6B: ii). This was similar to the binding mode of the **7_e_** compound with the **TMP** reference drug at the active site of the DHFR enzyme. The simulation interaction histogram of the investigated compounds is shown in iii panels of (Fig. 6) and demonstrates the comprehensive intermolecular contact profile of the **TMP** and **7_e_** compounds within the DHFR protein throughout the 100 ns simulation time. In addition, it showed that compound **7_e_** exhibited a preferable interaction fraction ratio. Apart from the docking results, the reference compound **TMP** demonstrated valuable H-bonding interaction with Asp27 amino acid (interaction fractio*n* = 90%), beside a water-bridged H-bonding with the same residue of about 50% fraction. Also, it displayed worthy H-bonding with the critical Phe31 residue (interaction fraction= 98%) throughout the simulation time (Fig. 6A: iii). In a similar manner, compound **7_e_** interacts with the Arg57 residue by H-bond with a significant interaction fraction of about 190%, along with a 20% ionic interaction, thereby proving the ultimate close contact through the whole simulation time. Besides, it revealed the considerable H-bonding interaction with the Arg52 residue of (interaction fraction= 100%), and a water-bridged H-bond with the same residue fraction (50%) (Fig. 6B: iii). The MD simulation data reveal without a doubt that the investigated compound **7_e_** in complex with the DHFR active site resided consistently during the 100 ns simulation.

### Suggested Structure-Activity Relationship

The structure-activity relationship showed that the replacement of the pyrimidine ring in **TMP** by the pyrazolo[3,4-*d*]pyrimidine nucleus bearing 4-aminobenzoic acid, followed by conjugation with several amino acids, enhances the DHFR inhibition activity in most of the prepared compounds. To be precise, glutamic acid is the most preferred amino acid conjugate as it possesses superior DHFR inhibition activity, as in compound **7_e_**. As a result, the glutamic acid in both α- and γ-carboxylate moieties is required for better orientation, scoring, and binding interaction with the critical residues at the DHFR active site to provide maximum DHFR inhibitory effect.

### Anti-QS and Anti-Virulence Activities of the Synthesized Compounds, Multi-Target Docking Analysis on QS Targets, and Docking Simulation on *P. aeruginosa* LasI-Type AHL Synthase and LasR QS Targets

The *in silico* study provided more insight into the anti-pseudomonal anti-virulence activity of the investigated pyazolo[3,4-*d*] pyrimidines **6** and **7_a-m_** towards two *P. aeruginosa* LasI/R QS systems. The *P. aeruginosa* LasI-type acyl-homoserine lactone (AHL) synthase (PDB ID: 1RO5) using **TZD-C8** as control, and the LasR (PDB ID: 6MVN), using **3MF** as native ligand, were employed in this study. Upon docking into the binding sites of LasI-Type AHL synthase, all of the investigated pyrazolo[3,4-*d*] pyrimidines **6** and **7_a-m_** revealed superior negative binding scoring over **TZD-C8** (-3.052 Kcal/mol), except compounds **7_i_** (-3.050 Kcal/mol) and **7_c_** (-2.996 Kcal/mol). Likewise, the purposed affinity of the investigated pyrazolo[3,4-*d*] pyrimidines towards the LasR active site were assessed. Fortuitously, all the trialed pyrazolo[3,4-*d*] pyrimidine compounds displayed higher binding energy than the **3MF** native ligand (-1.777 Kcal/mol) (Table 3).

### Docking Analysis of *S. aureus* ArgC Histidine Kinase ATP-Binding Domain

We rocked the ATP-binding domain of ArgC histidine kinase natural ligand **MPD** beside the scrutinized pyrazolo[3,4-*d*] pyrimidines compounds **6** and **7_a-m_** on the ArgC active site. Amazingly, we found that the normal ligand **MPD** demoed a lesser docking score of (-3.705 Kcal/mol). Auspiciously, all the investigated pyrazolo[3,4-*d*] pyrimidines show superior docking scores that exceeded the natural ligand **MPD**, except for compounds **7_c_** (-3.620 Kcal/mol) and **7_g_** (-3.325 Kcal/mol) (Table 3).

### *In Vitro* Anti-Virulence Activities of Promising Candidates

In light of the docking scores and compounds **6**, **7_a_**, **7_d_**, **7_e_**, and **7_k_** having shown considerable affinity to *S. aureus* QS-related AgrC, *P. aeruginosa* LasI synthetase, and/or its cognate LasR-QS receptor, the above compounds were selected for further evaluation of their anti-virulence activities against *S. aureus* and *P. aeruginosa*.

### Targeting Bacterial Virulence at Lower Concentrations (Sub-MIC) to Avoid Resistance Development

**Fig. 7:**
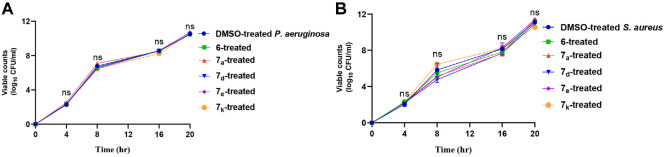
The tested compounds at (1/2 MIC) did not affect bacterial growth. There were no significant differences between (**A**) *P. aeruginosa* and (**B**) *S. aureus* counts in the presence or absence of selected compounds at 1/2 MIC. ns: nonsignificant *p* > 0.05.

QS systems play significant roles in controlling bacterial pathogenesis and synchronizing the expression of virulence factors [68-70]. These virulence factors include biofilm formation, proteins as virulent enzymes, toxins, and dyes that contribute to the capability of bacteria to establish infection and evade the host immune response [47, 68, 71]. Moreover, QS antagonism is an area of interest in the development of new antibacterial strategies aimed at interfering with bacterial communication and attenuate virulence [72-74]. This can be achieved by developing new synthetic compounds or natural products to act as competitive inhibitors, mimicking or blocking the binding sites of autoinducers, and preventing their recognition by bacterial receptors [10, 22, 35, 40]. Consequently, disrupting QS systems results in the reduction of bacterial virulence and limits their pathogenicity, facilitating the immune system's capability to eliminate infectious bacterial pathogens [21, 75]. This approach may complement traditional antibiotic treatments and address the growing challenge of antibiotic resistance. Ensuring this involves preventing any impact of the promising anti-virulence candidate on bacterial growth, without exerting stress that could lead to resistance development [16, 76, 77]. In this context, we tested the effect of the selected compounds at sub-MIC (1/2 MIC) on bacterial growth, and they showed no significant effect on the viable counts of *S. aureus* and *P. aeruginosa* (Fig. 7). The test was performed in triplicate and two-way ANOVA was used to detect statistical significance. QS systems are crucial for regulating the expression of various virulence factors in both gram-negative and gram-positive bacteria. [9, 78]. Regarding the virtual findings, which suggest a potential affinity of the synthesized compounds to QS targets in *S. aureus* and *P. aeruginosa*, those with the highest score were **6**, **7_a_**, **7_d_**, **7_e_**. Compound **7_k_** underwent additional anti-virulence studies.

### Anti-Biofilm Activity

QS is crucial for the formation of biofilms, which are structured communities of bacteria encased in a protective matrix [69, 79]. Biofilms exhibit increased resistance to antibiotics and immune responses compared to planktonic (free-floating) bacteria. The biofilm matrix acts as a physical barrier that limits the penetration of antimicrobial agents and shields bacteria from the host immune system [80, 81]. Biofilms are associated with chronic and persistent infections as bacteria within biofilms can evade host defenses, making it a challenge for the immune system to completely clear the infection [82, 83]. Biofilms enhance bacterial survival in diverse environments, allowing bacteria to persist in various niches within the host or the external environment [44, 84]. In addition, biofilms can facilitate horizontal gene transfer among bacteria, promoting the exchange of genetic material and potentially enhancing the adaptability of bacterial populations [85-87].

**Fig. 8:**
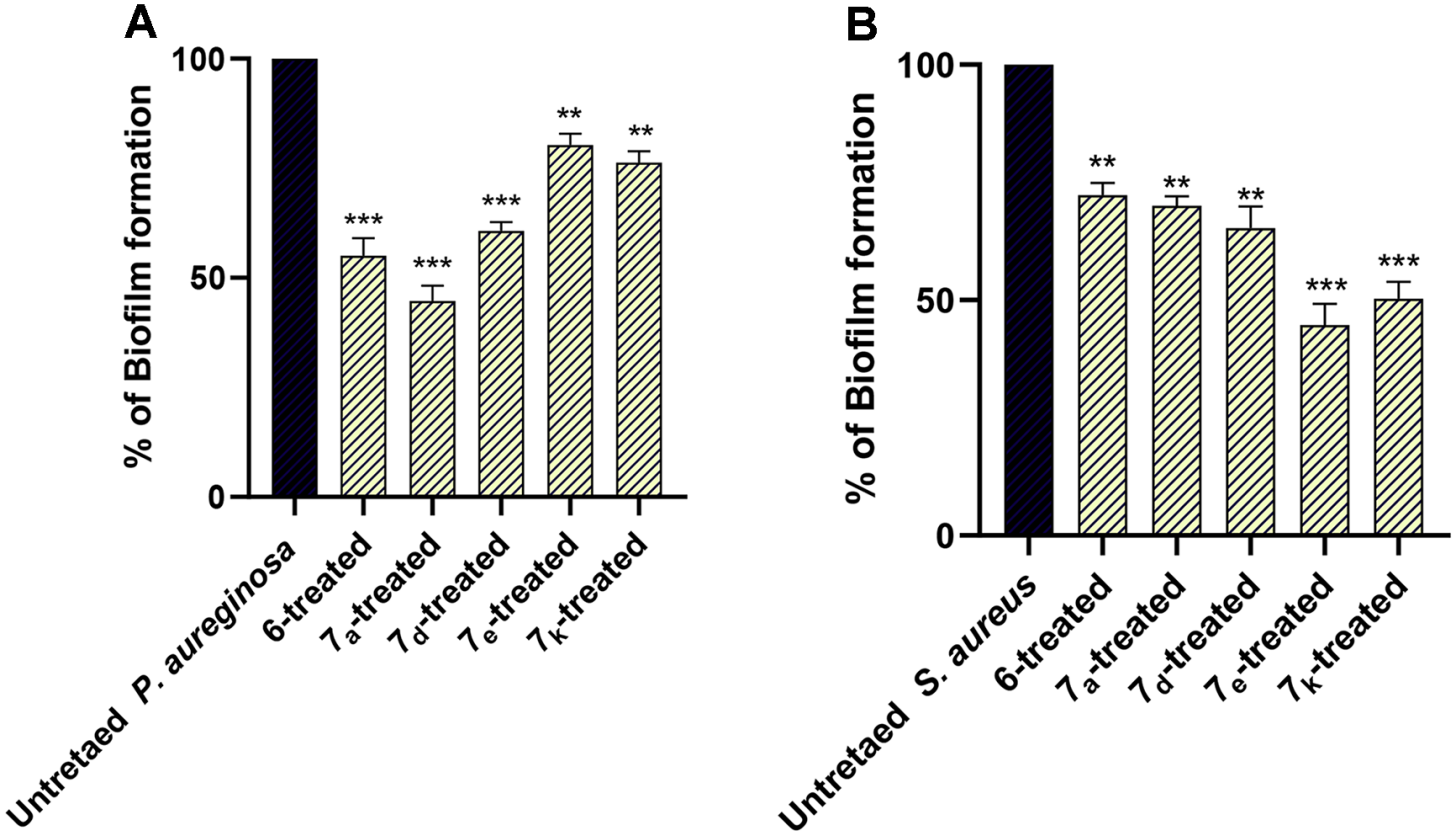
The antibiofilm activity of the selected compounds at sub-MIC (1/2 MIC). The biofilm formation in (**A**) *P. aeruginosa* and (**B**) *S. aureus* was significantly diminished in the presence of the compounds at sub-MIC, ** *p* < 0.01; *** *p* < 0.001.

The current results showed the prominent ability of the selected compounds at sub-MIC (1/2 MIC) to inhibit biofilm formation. The compounds significantly diminished biofilm formation by *P. aeruginosa* and *S. aureus*, while compounds **6**, **7_a_**, and **7_d_** were more efficient against *P. aeruginosa*, and compounds **7_e_** and **7_k_** were more efficient against *S. aureus* (Fig. 8). These results are in great compliance with the docking scores and highlight the potential anti-biofilm activity of the tested compounds. The experiment was performed in triplicate, and statistical significance was determined using a one-way ANOVA. The presented data were calculated as a percent change from untreated bacterial control.

### Inhibition of Virulence Factors

Bacterial virulence factors contribute to the remarkable ability of bacteria to cause infection and disease in a host. These factors enhance the pathogenicity of bacteria by promoting their survival, adhesion to host tissues, evasion of immunity, and damage to host cells [88, 89]. Bacteria often employ a combination of virulent extracellular enzymes, including proteases and hemolysins, to establish and maintain infections [89, 90]. Proteases are enzymes that cleave host proteins into smaller peptides or amino acids. They play a crucial role in various stages of infection, contributing to the degradation of host tissues and facilitating the spread of bacteria within the host [91]. Proteases assist in acquiring nutrients by breaking down host proteins into amino acids that can be utilized by bacteria for growth [91, 92]. Furthermore, proteases can degrade antibodies and other components of the immune system, allowing bacteria to evade immune detection [93, 94]. On the other hand, hemolysins are toxins that cause the lysis (rupture) of red blood cells, which contributes to tissue damage and nutrient acquisition [95, 96]. Proteases and hemolysins are key contributors to the overall pathogenicity of bacteria. They aid in tissue invasion, immune evasion, and nutrient acquisition, so targeting them could therefore lessen bacterial virulence [16, 76, 97].

**Fig. 9:**
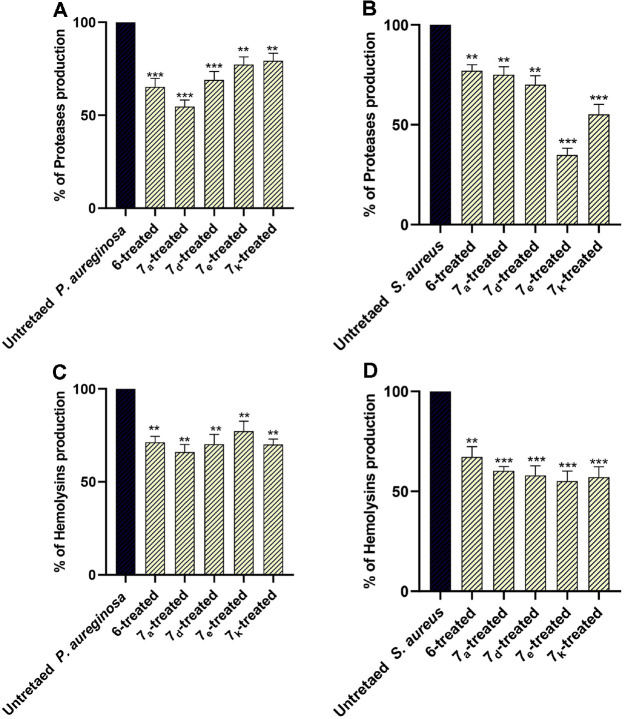
The inhibitory effect of the selected compounds at sub-MIC (1/2 MIC) on the production of proteases in (**A**) *P. aeruginosa* and (**B**) *S. aureus*, and hemolysins in (**C**) *P. aeruginosa* and (**D**) *S. aureus*. The compounds at sub-MIC significantly lowered the production of proteases and hemolysins, ** *p* < 0.01; *** *p* < 0.001.

Likewise, QS systems regulate the release of proteases and hemolysins, and as a result, antagonizing QS could lead to a decrease in their production [68, 98]. In alignment with the virtual findings that showed the potential anti-QS activity of the selected compounds, these compounds significantly decreased the protease and hemolysin production in *P. aeruginosa* and *S. aureus* (Fig. 9). The experiments were done in triplicate, and the findings are depicted as percentage change compared to untreated bacterial controls. Statistical significance was evaluated using one-way ANOVA.

### *In Vivo* Mice Protection

**Fig. 10:**
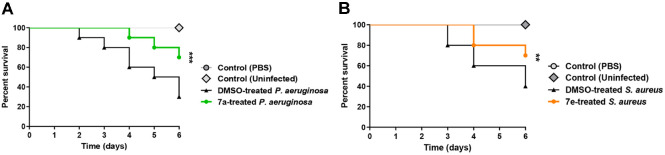
The *in vivo* protection activity of (**A**) compound 7_a_ at 1/2 MIC against *P. aeruginosa* and (**B**) compound 7_e_ at 1/2 MIC against *S. aureus*. Compound **7_a_** protected mice from *P. aeruginosa* decreasing the number of deaths from 7 to 4 out of 10 mice (log rank test for trend *p* = 0.0002). Moreover, **7_e_** protected mice against *S. aureus* lowering the dead mice to 3 instead of 6 out of 10 mice in the positive control group (log-rank test for trend *p* = 0.0026), ** *p* < 0.01; *** *p* < 0.001.

To establish the anti-virulence activity of these compounds, a mice protection assay was conducted against *S. aureus* and *P. aeruginosa*. In light of our in silico and *in vitro* anti-virulence findings, compounds **7_a_** and **7_e_** were chosen for evaluation of their *in vivo* anti-virulence activities against *P. aeruginosa* and *S. aureus*, respectively (Fig. 10). While there was no observed death in the negative control groups, the deaths were 7 out of 10 in the mice group injected with DMSO-treated *P. aeruginosa*, and 6 out of 10 in the mice group injected with DMSO-treated-*S. aureus*. The number of recorded deaths was reduced to 4 out of 10 in the mice group that was injected with *P. aeruginosa* treated with **7_a_** at 1/2 MIC. In the mice group that was injected with *S. aureus* treated with **7_e_** at 1/2 MIC, the deaths were 3 out of 10. These results indicate the significant ability of compounds **7_a_** and **7_e_** to diminish the capacity of *P. aeruginosa* and *S. aureus* to induce pathogenesis in mice (log-rank test for trend *p* = 0.0002 and 0.0026, respectively).

### *In Silico* Interactions of the Most Active Anti-Virulence Candidates against QS Biotargets and Virtual Interaction of Compound 7_a_ with LasI-Type AHL Synthase Target

**Fig. 11:**
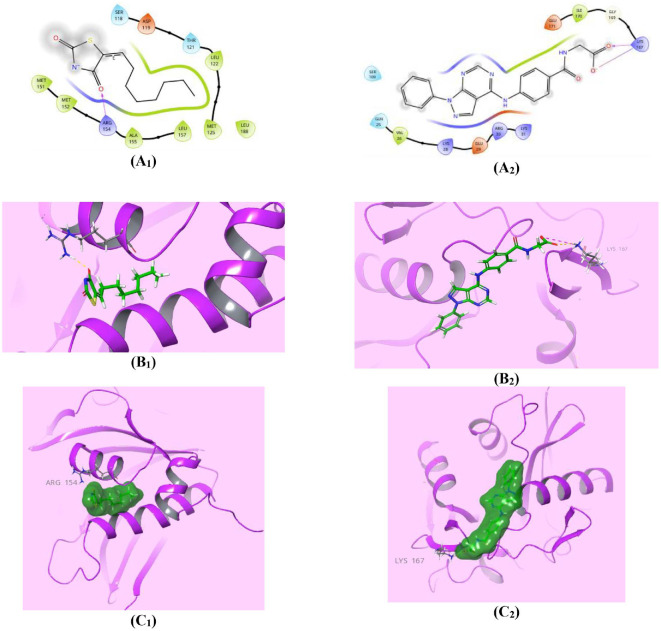
The binding interactions between compound 7_a_ and LasI-synthetase. (**A_1,2_**) Two-dimensional structural representation of the reference ligand control (TZD-C8) and the investigated pyrazolo[3,4-*d*]pyrimidine derivative **7_a_** in the active site of *P. aeruginosa* LasI-type AHL synthase (pdb ID: 1RO5). (**B_1,2_**) Three-dimensional representation of the *P. aeruginosa* LasI-type AHL synthase binding site with an overlay of the reference ligand control (TZD-C8) and the pyrazolo[3,4- d]pyrimidine derivative **7_a_** (green sticks). (**C_1,2_**) Three-dimensional cartoon representations of the *P. aeruginosa* LasI-type AHL synthase binding domain quorum-sensing transcription proteins co-crystallized with the reference ligand control (TZD-C8) and the pyrazolo[3,4-*d*]pyrimidine derivative **7_a_** (green space-filling).

In correspondence with the *in vitro* assay results, compound **7_a_** bearing acyl glycine scaffold revealed a better LasI-type AHL synthase-binding interaction score (-4.277 *K*_cal_/mol) than the reference control *TZD-C8* (*S* = -3.052 Kcal/mol). The favorable binding affinity of compound **7_a_** may be attributed to the valuable ionic interaction of the terminal carboxylate moiety with the crucible Lys167 residue (4.40A^o^) beside the H-bonding acceptor effect of the carbonyl group of the same carboxylate with the same residue (2.04A^o^). Meanwhile, the reference control **TZD-C8** only showed an H-bonding acceptor effect of the 4-carbonyl group of the thiazolidine ring with the Arg154 residue (1.81A^o^) (Fig. 11A_1,2_, 11B_1,2_, and 11C_1,2_). Significantly, the above interactions of Lys167 residue in ionic binding with compound **7_a_** carboxylic group play an important role in forming a highly positively charged patch on the LasI surface during its binding to the acyl carrier, and require further investigation [49].

### Virtual Interaction of Compound 7_a_ with LasR-Type Target

**Fig. 12:**
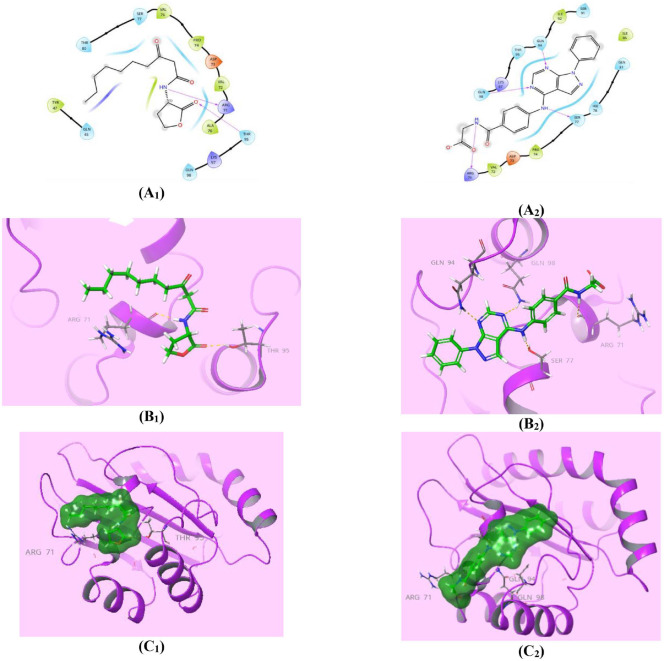
The binding interactions of compound 7_a_ to LasR QS receptor. (**A_1,2_**) Two-dimensional structural representation of the reference ligand **3MF** and the investigated pyrazolo[3,4-*d*]pyrimidine derivative **7_a_** in the active site of *P. aeruginosa* LasR-type (pdb ID: 6MVN). (**B_1,2_**) Three-dimensional representation of the *P. aeruginosa* LasR-type binding site with an overlay of the reference ligand **3MF** and the pyrazolo[3,4-*d*]pyrimidine derivative **7_a_** (green sticks), where the amino acid residues (grey lines) located within 5 Å radius distance from the bound ligand and labeled with sequence number. (**C_1,2_**) Three-dimensional cartoon representations of *P. aeruginosa* LasR-type binding domain quorum-sensing transcription proteins co-crystallized with the reference ligand **3MF** and the pyrazolo[3,4-*d*]pyrimidine derivative **7_a_** (green space-filling).

The LasR reference ligand **3MF** (*S* = -1.777 Kcal/mol) performed as a substantial H-bond donor between its amidic N-H group and the crucible Arg71 amino acid (1.95A^o^), in addition to being a significant H-bond acceptor of the lactone carbonyl group with Thr95 (1.77A^o^) (Fig. 12A_1_, 12B_1_, and 12C_1_). However, the inspected pyrazolo[3,4-*d*] pyrimidine bearing acyl glycine compound **7_a_** anchored accurately almost at the same orientation as the reference ligand with higher affinity to the LasR active site of (*S* = -6.156 Kcal/mol), owing to two characteristic H-bond acceptor interactions between both pyrimidine nitrogen with Gln94 (2.03A^o^) and Gln98 (2.16A^o^) residues. Moreover, the presence of these two protruding H-bond donor effects is due to the secondary amino group with the Ser77 residue (2.06A^o^), and the secondary amidic N-H group with the Arg71 amino acid (2.50A^o^), as shown in (Fig. 12A_2_, 12B_2_, and 12C_2_). Thus, the pyrazolo[3,4-*d*] pyrimidine bearing acyl glycine compound **7_a_** can be seen to exhibit conceivable binding interaction with LasR-Type quorum-sensing proteins with expected anti-virulence activity against *P. aeruginosa*.

### Virtual Interactions of Compound 7_e_ with ArgC Histidine Kinase ATP-Binding Domain

**Fig. 13:**
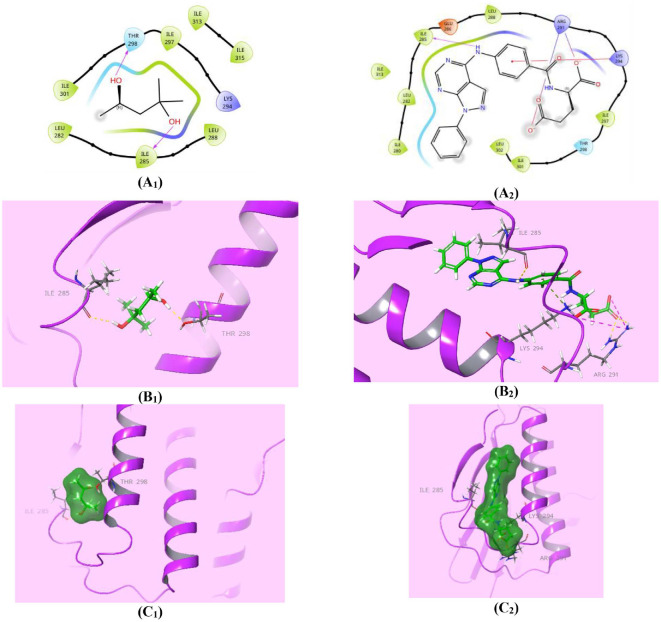
The binding interactions of compound 7_e_ with *S. aureus* AgrC. (**A_1,2_**) Two-dimensional structural representation of the reference ligand **MPD** and the investigated pyrazolo[3,4-*d*]pyrimidine derivative **7_e_** in the active site of ATP-binding domain of *S. aureus* AgrC histidine kinase. (pdb ID: 4BXI). (**B_1,2_**) Three-dimensional representation of the ATPbinding domain of *S. aureus* AgrC histidine kinase with an overlay of the reference ligand **MPD** and the pyrazolo[3,4- d]pyrimidine derivative **7_e_** (green sticks), where the amino acid residues (grey lines) located within 5 Å radius distance from the bound ligand and labeled with sequence number. (**C_1,2_**) Three-dimensional cartoon representations of *S. aureus* AgrC histidine kinase binding domain quorum-sensing transcription proteins co-crystallized with the reference ligand **MPD** and the pyrazolo[3,4-*d*]pyrimidine derivative **7_e_** (green space-filling).

The higher binding affinity of compound **7_e_** (*S* = -5.322 Kcal/mol) may be attributable to its deep anchoring at the active site and high ligand exposure to numerous residues, despite the helices steric shield. Also, it accomplishes noteworthy ionic bonding of both its α- and γ-carboxylates of the glutamic acid with the Arg291 residue of bond length (4.89A^o^) and (4.56A^o^), respectively. Additionally, it revealed characteristic π-cation interaction with the Lys294 residue (3.54A^o^) along with characteristic H-bond donor effect of its secondary amino group with the critical Ile285 amino acid (2.11A^o^) (Figs. 13A_2_ and 4B_2_). Correspondingly, the native ligand **MPD** exerted two characteristic H-bond donor effects at the same region; one interaction with the crucial Ile285 (2.13A^o^), and the other with the Thr298 residue (1.97A^o^), but with lower binding affinity of (*S* = -3.705 Kcal/mol). Hence, compound **7_e_** could generally be adopted as a potential anti-virulence agent against *S. aureus* through inhibition of the ATP-binding domain of ArgC histidine kinase, but requires further precision.

### MD Simulation of the Most Active Anti-Virulence Candidates against QS Biotargets

We performed a molecular dynamic simulation lasting 100 ns to consider the dynamic behavior of the quorum sensing (QS) biotargets; LasI-synthetase and LasR-type in complex with the most active pyrazolo[3,4-*d*] pyrimidine incorporating glycine **7_a_**, and related to the native ligands **TZD-C8** and **3MF**, respectively. In addition, we measured the stability of the complex between the AgrC histidine kinase active site with compound **7_e_** compared to the **MDP** native ligand.

### MD Simulation of Compound 7_a_ in Complex with the LasI-Type AHL Synthase Related to TZD-C8 Control

**Fig. 14:**
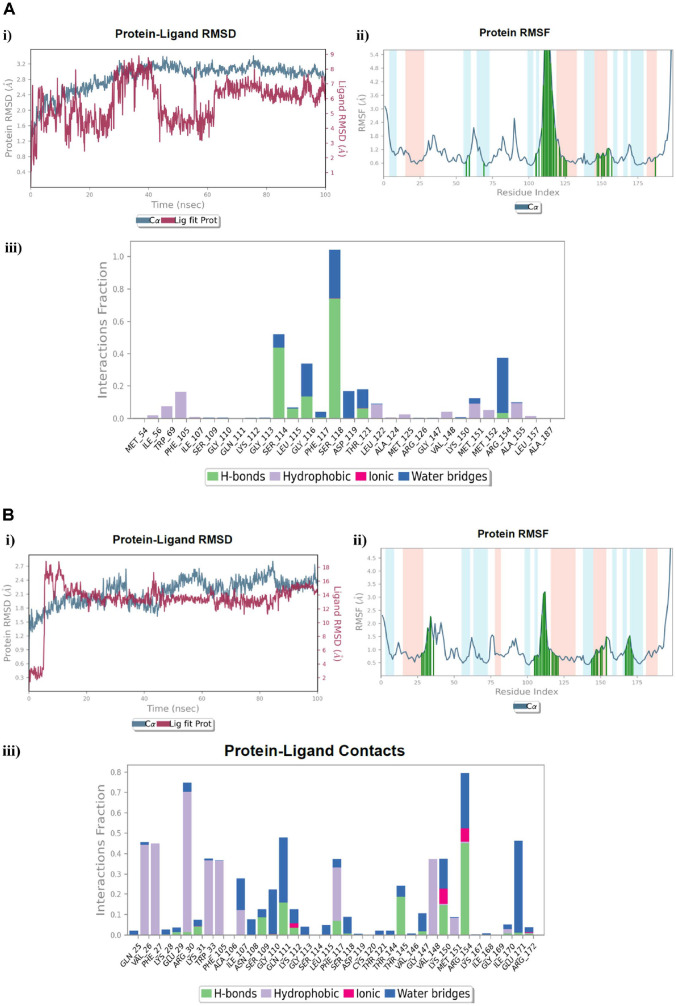
MD simulation analysis of; (**A**) The ligand control **TZD-C8** in complex with the LasI-synthetase active site, (**B**) The investigated compound **7_a_** in complex with the LasI-synthetase active site, **i**) RMSD (Protein RMSD is shown in grey while the RMSD of the **TZD-C8** and **7_a_** ligands is shown in red), **ii**) Protein RMSF, **iii**) Protein–ligand contact histogram analysis of the MD trajectory.

Inappropriately, the protein RMSD exploration of the LasI-type AHL synthase complex with the **TZD-C8** control revealed that the Cα atom distinctions were consistently within 3.2 Å throughout the simulation and exceeded the allowed limit of 3.0 Å (Fig. 14A: i). However, regarding the protein RMSD analysis of the LasI-type AHL synthase complex with compound **7_a_**, the Cα atom variations were regularly within the acceptable limit at 2.9 Å, which manifests the approximate priority of the binding behavior of compound **7_a_** over the control **TZD-C8** (Fig. 14B: i). Moreover, the RMSD of the **TZD-C8** and **7_a_** ligands after the fluctuation equilibrium were between 3.0 Å and 9.0 Å, and from 12.0 Å to 18.5 Å, respectively. Regarding the protein RMSF layout, both the **TZD-C8** control and the inspected **7_a_** compound shared contact with 16 residues of the LasI-type AHL synthase protein, with most of them located at the loop region, namely Phe105, Ile107, Ser109, Gly110, Gln111, Lys112, Gly113, Ser114, Leu115, Phe117, Ser118, Asp119, Thr121, Lys150, Met151, and Arg154. Nonetheless, the pyrazolo[3,4-*d*]pyrimidine compound **7_a_** displayed favorable contact with an additional 20 residues, namely Gln25, Val26, Phe27, Lys28, Glu29, Arg30, Lys31, Trp33, Ala106, Asn108, Cys120, Thr144, Thr145, Val146, Lys167, Ile168, Gly169, Ile170, Glu171, and Arg172, with most of them being at the loop and β-pleated sheet regions (Fig. 14A, 14B: ii). Meanwhile, the ligand-protein contact histogram revealed that the **TZD-C8** control combined with Ser118 by H-bonding and an interaction fraction of about 78%, and the remaining 15% represented a water-bridged H-bond with the same residue. Also, it demonstrated a 45% H-bonding interaction fraction with the Ser114 residue along with a water-bridged H-bond of about 10% (Fig. 14A: iii). Beneficially, compound **7_a_** revealed higher interactions with numerous additional residues, including favorable contact with protein, such as the Agr154 residue by H-bonding (45%), ionic (10%), and water-bridged H-bond (25%). It also had contact with the Arg30 residue by hydrophobic interaction (70%) and water-bridged H-bond (5%) (Fig. 14B: iii).

### MD Simulation of Compound 7_a_ in Complex with the LasR-Type Target Related to 3MF Ligand

**Fig. 15:**
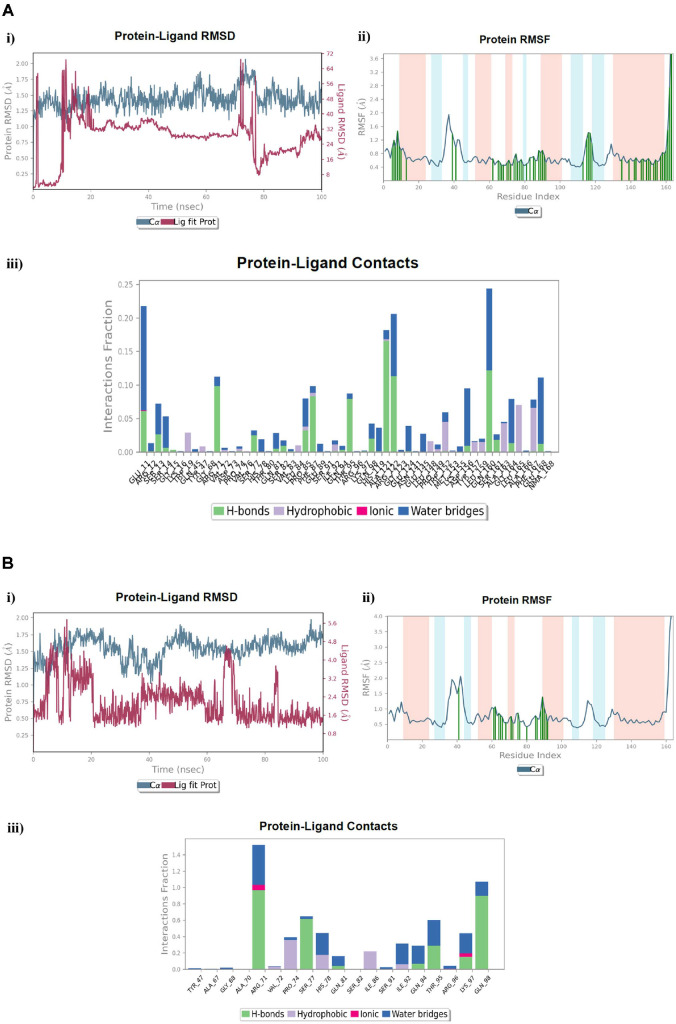
MD simulation analysis of; (**A**) The ligand control **3MF** in complex with the LasR-type QS active site, (**B**) The investigated compound **7_a_** in complex with the LasR-type QS active site, **i**) RMSD (Protein RMSD is shown in grey while the RMSD of the **3MF** and **7_a_** ligands is shown in red), **ii**) Protein RMSF, **iii**) Protein–ligand contact histogram analysis of the MD trajectory.

Examination of the protein RMSD of the LasR-type complex with **3MF** ligand showed that the Cα atom divisions were consistently within 2.0 Å throughout the simulation, while they retracted to beyond 2.0 Å in the **7_a_**-LasR-type complex, which reflected less fluctuation and more stable protein during the MD simulation involving **7_a_** compound. On another hand, the RMSDs of the **3MF** and **7_a_** ligands, after steadiness of the fluctuations was achieved, were between 30.0 Å and 70.0 Å, and from 1.4 Å to 4.8 Å, respectively, thereby demonstrating the lower fluctuation and greater stability of the **7_a_**-LasR-type complex (Fig. 15A, 15B: i). In addition, both compounds **3MF** and **7_a_** participated in combining 16 residues during the simulation time upon examination of the protein RMSF. The residues were Tyr47,Gly68, Arg71, Val72, Pro74, Ser77, His78, Gln81, Ser82, Ser91, Ile92, Gln94, Thr95, Arg96, Lys97 and Gln98. Moreover, compound **7_a_** exhibited 3 diverse interactions with the hydrophobic Ala67, Ala70, and Ile86 residues (Fig. 15A, 15B: ii). Although the **3MF** ligand contacted a greater number of residues during the simulation, the ligand-contact histogram revealed that lower ratio of interaction fraction did not exceed 25% (Fig. 15A: iii). Correspondingly, and in parallel with the docking assessment results, compound **7_a_** contacted the following residues with elevated interaction fraction ratios: 1. Agr71 residue by H-bond (95%), ionic bond (5%) and water-bridged H-bond (45%), 2. Gln98 residue by H-bond (90%), and water-bridged H-bond (20%) (Fig. 15B: iii). These results demonstrated the stability of the **7_a_**-LasR complex during the simulation and accounted for the predicted efficacy.

### MD Simulation of Compound 7_e_ in Complex with the ArgC Histidine Kinase ATP-Binding Domain Related to MPD Ligand

**Fig. 16:**
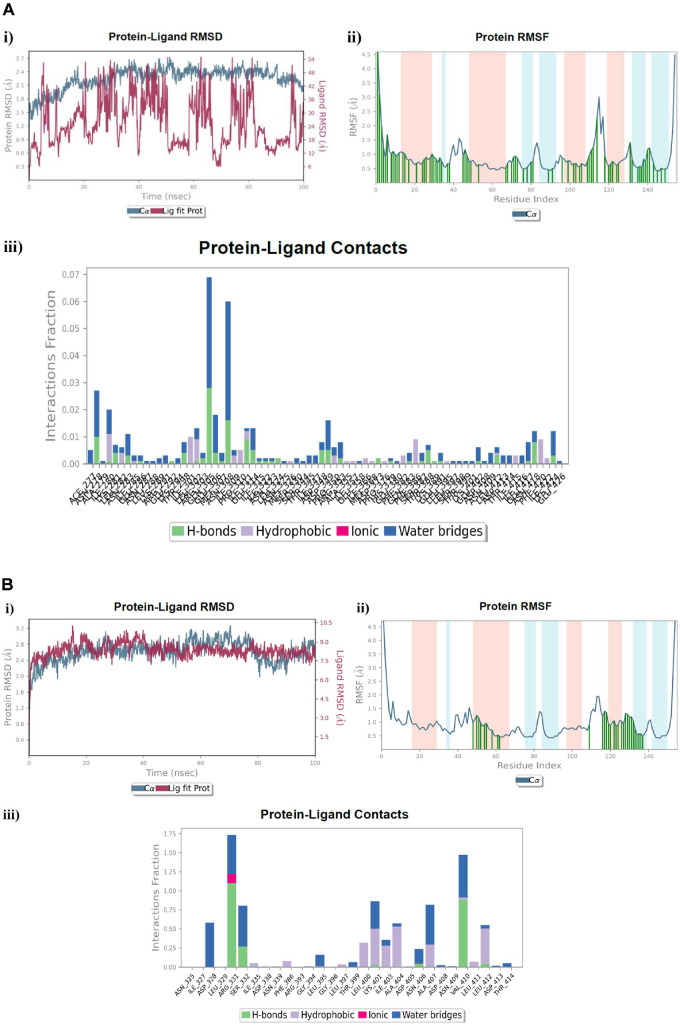
MD simulation analysis of; (**A**) The ligand control **MPD** in complex with the ATP-binding domain of AgrC histidine kinase, (**B**) The investigated compound **7_e_** in complex with the the ATP-binding domain of AgrC histidine kinase, **i**) RMSD (Protein RMSD is shown in grey while the RMSD of the **MPD** and **7_e_** ligands is shown in red), **ii**) Protein RMSF, **iii**) Protein–ligand contact histogram analysis of the MD trajectory.

Although the Cα atom dissections during the simulation of the **MPD**-ArgC histidine kinase complex were constantly within 2.7 Å and did not exceed the normal ratio, the protein RMSD of the complex with the investigated **7_e_** compound revealed deviation variations to 3.3 Å. In contrast, the ligand RMSD of the **7_e_** compound after equilibrium was between 7.5 Å and 10.5 Å, where it remained between 6.0 Å to 54.0 Å for the **MPD** native ligand, authorizing fewer fluctuations of compound **7_e_** throughout the 100 ns fluctuation time (Fig. 16A, 16B: i). The protein RMSF of both compounds **MPD** and **7_e_** revealed contact with 11 residues, namely Asn325, Phe386, Leu395, Leu397, Thr399, Lys401, Asp408, Asn409, Leu412, Asp413, and Thr414. Meanwhile, compound **7_e_** exhibited 19 unique interactions with the residues Ile327, Asp328, Leu329, Arg331, Ser332, Ile335, Asp338, Asn339, Arg393, Gly394, Glu396, Leu400, Ile403, Ala404, Asp405, Asn406, Ala407, Val410, and Leu411 (Fig. 16A, 16B: ii). Furthermore, although the **MPD** complex combined with numerous residues, the ligand-protein contact histogram revealed an inferior interaction fraction ratio that did not exceed 7%. In contrast, for the investigated compound **7_e_**, the histogram showed vibrant interaction with ratios and contact as follows: Agr331 residue by H-bond (110%), ionic bond (12%), and water-bridged H-bond (50%); Val410 residue by H-bond (95%), and water-bridged H-bond (500%) (Fig. 16B: iii). The resulting outcomes were in agreement with the stability of the **7_e_**- ArgC histidine kinase complex throughout the simulation time but will require further biological assessment.

## Conclusion

The present study showed promising antibacterial activity for pyrazolo[3,4-*d*] pyrimidine-based analogues through targeting the DHFR enzyme, in addition to a prominent anti-virulence effect against *P. aeruginosa* and *S. aureus* strains. These compounds displayed a potent diminishing effect on the biofilm formation and production of virulent factors, such as proteases and hemolysins. The structural features of the most potent candidates involve the replacement of the pyrimidine ring in **TMP** by a pyrazolo[3,4-*d*] pyrimidine core bearing 4-aminobenzoic acid, which enhances both the antibacterial and anti-virulence effects. Conjugation of pyrazolo[3,4-*d*]pyrimidine analogues with the hydrophilic residue glutamic acid exhibits superior DHFR inhibition activity and anti-virulence effect against *S. aureus* ArgC histidine kinase, as in compound **7_e_**. Moreover, conjugation with the hydrophobic glycine residue with the pyrazolo[3,4-*d*]pyrimidine nucleus in compound **7_a_** demonstrated extensive anti-virulence effects against *P. aeruginosa* LasI- and LasR-type biotargets that require further investigation. This study also demonstrated the *in vitro* and *in vivo* activities, particularly of compounds **7_a_** and **7_e_** against *P. aeruginosa* and *S. aureus*, respectively, suggesting their potential for controlling aggressive bacterial infections, either alone or in combination with antibiotics. However, our promising findings and the clinical employment of these compounds will require further toxicological and pharmaceutical investigations.

## Supplemental Materials

Supplementary data for this paper are available on-line only at http://jmb.or.kr.



## Figures and Tables

**Table 1:** MICs in μg/ml against standard strains of diverse bacterial strains.

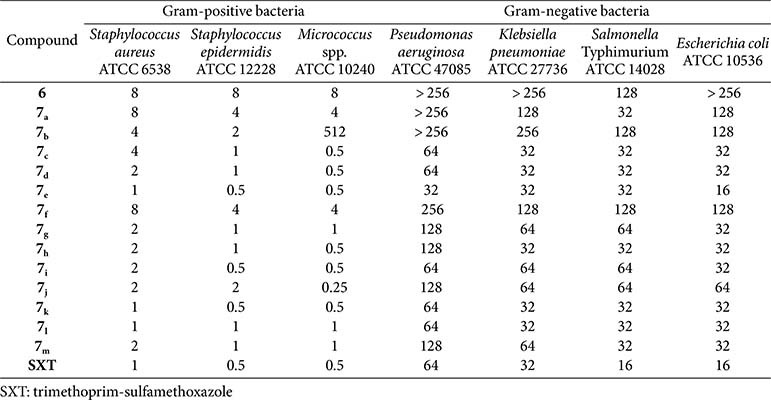

**Table 2:** The calculated parameters of Lipinski’s rule for the investigated pyrazolo[3,4-*d*]pyrimidine compounds 6, and 7_a-m_, including molecular weight (MW), partition coefficient (LogP), number of hydrogen bonding donors (nHBD), number of hydrogen bonding acceptors (nHBA) and the druglikeness.

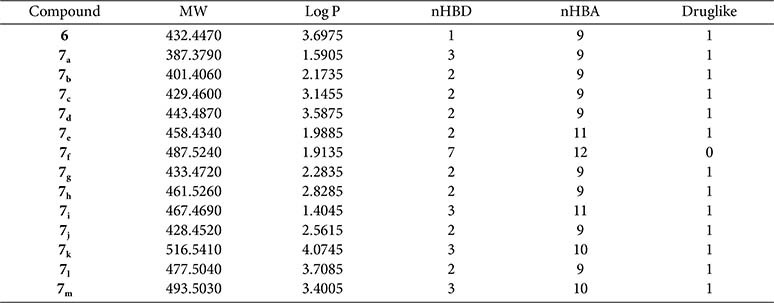

**Table 3:** Docking scores (- Kcal/mol) of the investigated pyrazolo[3,4-*d*]pyrimidine compounds 6, 7_a-m_ and the selected ligands **TZD-C8**, 3MF, and **MPD** against the active site of *P. aeruginosa* LasI-synthetase (PDB: 1RO5), LasR-type (PDB: 6MVN), and the ATP-binding domain of *S. aureus* AgrC histidine kinase (PDB: 4BXI).