# Supplementary material for: Disclosing New Insights on Pyrazolo[3,4-d] Pyrimidine Tethered Diverse Amino Acid Candidates as Potential DHFR Inhibitors and Anti-Virulence Agents
Source: J Microbiol Biotechnol. 2025 Dec 11;35:e2508055. doi: 10.4014/jmb.2508.08055 (PMC12706152; doi:10.4014/jmb.2508.08055)
Supplement: Supplementary file 1 [file jmb-35-e2508055-supple.pdf]

J. Microbiol. Biotechnol. 2025. 35: e2508055
https://doi.org/10.4014/jmb.2508.08055

Disclosing new insights of pyrazolo[3,4-*d*]pyrimidines tethered diverse amino acid candidates as potential DHFR inhibitors and anti-virulence agents

Tarek S. Ibrahim[1,*], Ibrahim M. Salem [2,3], Nabil A. Alhakamy[4,5,6], Amany M. M. Al-Mahmoudy [7], Wael A. H. Hegazy [8,9]

Supplementary Data

1. **Detection of Mininmum inhbitory concentration**

The MICs of tested compounds were detected utilizing the broth microdilution method following the directives outlined by the Clinical Laboratory and Standards Institute (CLSI, 2020). Two-fold serial dilutions of tested compounds from 0.03 to 512 μg/mL were prepared in Mueller–Hinton broth. One hundred μl aliquots were transferred ito the microtiter plates to be mixed with equal volume of bacterial suspensions with approximate density 1×10^6 CFU/mL. Following an overnight incubation of the microtiter plate at 37°C, the wells were inspected for growth, and the MIC was identified as the lowest concentration that prevented visible bacterial growth.

2. **DHFR inhibition assay**

Dihydrofolate Reductase Inhibitor Screening Kit (Cat No. ab283374, Abcam, MA, USA) was used to screen the DHFR inhibitors. The positive and negative controls and the samples of compounds (at MIC concentrations) were prepared according to the protocol provided by the manufacturer. The activity of DHFR is observed by the decrease in absorbance at OD 340 nm, while potential inhibitors could arrest this decrease.

The used protcol accoerding manfacturer as follow:

Before using the kit, spin the tubes prior to opening. DHFR Assay Buffer: DHFR Substrate: Warm to room temperature (RT) before use. Store at 4°C or -20°C. Aliquots and store at -80°C, protect from light. Avoid repeated freeze/thaw. Dihydrofolate Reductase: Supplied as liquid. Divide into aliquots and store at -20°C. Avoid repeated freeze/thaw cycles. Keep on ice while in use. NADPH: Reconstitute the vial in 200 μL DHFR Assay Buffer to generate NADPH Stock Solution. Divide into aliquots and store at -20°C. Keep on ice while in use. Methotrexate (10 mM): Divide into aliquots and store at -20°C.

Dilute Methotrexate 100-fold (i.e. Dilute 2 μl Methotrexate with 198 μl DHFR Assay Buffer). Dissolve test sample to 100X in an appropriate solvent. Add 2 μl of the test sample, DHFR Assay Buffer or Diluted methotrexate into wells assigned as Sample Screening (S), Enzyme Control (EC) or Inhibitor Control (IC), respectively. Dilute Dihydrofolate Reductase 400-fold (i.e. Dilute 2 μl Dihydrofolate Reductase with 798 μl DHFR Assay Buffer). Prepare enough enzyme mix for the number of wells to be analyzed. Add 98 μl of diluted Dihydrofolate Reductase into desired well(s) containing the test samples, Enzyme Control or Inhibitor Control. The partial volume is 100 μl. Add 100 μl DHFR assay buffer to desired well(s) as Background Control. Prepare a 40-fold dilution of the NADPH stock solution (i.e. Dilute 10 μl of NADPH stock solution with 390 μl DHFR Assay Buffer), vortex briefly and keep on ice. Add 40 μl of diluted NADPH to each well containing the test samples, Enzyme Control, Inhibitor Control or Background Control. Mix well. Incubate at room temperature for 10-15 min, avoid light. Prepare a 15-fold dilution of DHFR Substrate (i.e. Dilute 40 μl of DHFR stock Substrate with 560 μl DHFR Assay Buffer), vortex briefly and keep on ice. Add 60 μl of diluted DHFR Substrate to each well containing the test samples, Enzyme Control, Inhibitor Control or Background Control. Mix well. The total volume should be 200 μl.
Finally, Measure absorbance immediately at 340 nm in kinetic mode for 10-20 min at RT. Choose any two time points (t1 & t2) in the linear range of the plot and obtain the corresponding values for the absorbance (OD1 and OD2). Calculate the slope for all test Inhibitor Samples [S] & Enzyme Control [EC] by dividing the net ΔOD (A1-A2) values with the time Δt (t2-t1). Subtract the Solvent Control or Inhibitor Background Control readings from its paired sample readings. % Relative Inhibition = $slope\ of [EC] – slope\ of\ [S] / slope\ of\ [EC]$ ×100 and % Relative activity = $slope\ of\ [S]\ /\ slope\ of\ [EC]$ ×100.

3. **Viable counting to evaluate effect of tested compounds at sub-MIC on bacterial growth**
Broth, with or without the tested compound at 1/2 MIC, was inoculated with a standard bacterial suspension to a final density of 1 × 10^6 CFU/mL. Samples were collected at 4, 8, 16, and 20-hour intervals, serially diluted, and plated onto Mueller-Hinton agar. The viable bacterial count was determined by enumerating the resulting colonies and is reported as Colony Forming Units per milliliter (CFU/mL).
